# Food availability and advertising within food outlets around primary healthcare services in Brazil

**DOI:** 10.1017/jns.2020.45

**Published:** 2020-11-11

**Authors:** Paula M. Horta, Juliana de P. M. Souza, Patrícia P. Freitas, Aline C. S. Lopes

**Affiliations:** Departamento de Nutrição, Universidade Federal de Minas Gerais, Belo Horizonte, Minas Gerais, Brazil

**Keywords:** Food marketing, Ultra-processed foods, Fruit and vegetables, Food consumer environment

## Abstract

The consumer food environment is changing: an extensive variety of foods are now available in most markets, offering palatability, convenience and novelty. However, little is known about the availability and advertising of food items within food outlets, especially among developing countries. The present study examined these dimensions in 281 food outlets located around eighteen primary healthcare services in Belo Horizonte, Brazil, in 2013. These establishments were classified as large-chain supermarkets; specialised fruits and vegetable (F&V) markets; and local grocery stores, convenience stores or bakeries. Availability of F&V, availability of ultra-processed foods (UPF) and food advertising were compared across the food outlet categories by applying the *χ*^2^ test. Almost 60 % of the food outlets were specialised F&V markets, 21⋅4 % were large-chain supermarkets and 19⋅2 % were local grocery stores, convenience stores or bakeries. Almost 80 % contained at least eight types of fruits and vegetables, and 60 % contained UPF. Food advertisement was absent in 59⋅8 % of the food outlets, 19⋅6 % were advertising only F&V and 17⋅4 % were advertising only UPF. Higher F&V availability was noted inside specialised F&V markets and large-chain supermarkets than local grocery stores, convenience stores or bakeries. Advertising of F&V was more common within specialised F&V markets. However, large-chain supermarkets and local grocery stores, convenience stores or bakeries contained more frequent UPF food advertising isolated: 38⋅3 and 35⋅2 %, respectively. Therefore, the availability and advertising of food items within food outlets around primary healthcare services are different according to the type of food outlet.

## Introduction

Eating habits are influenced by a combination of individuals factors and characteristics of the food environment, such as the availability, accessibility, affordability, desirability, convenience, marketing, and the properties of food sources and products^(1–4)^. The International Network for Food and Obesity/Non-Communicable Diseases (NCDs) Research, Monitoring and Action Support (INFORMAS) is a global network of public interest organisations and researchers from different countries that aims to monitor, benchmark, and support public and private sector actions to increase healthy food environments and reduce obesity, diet-related NCDs, and their related inequalities^(5)^. INFORMAS developed modules for which the monitoring frameworks have been designed and the indicators determined, including the food retail environment module^(6)^.

The food retail environment can be divided into the community food environment (the type, availability and accessibility of food outlets) and consumer food environment (the availability, prices, promotions and nutritional quality of products available within stores)^(6,7)^. The food retail environment is changing: an extensive variety of food and beverages are now available in most markets, offering palatability, convenience and novelty. However, at the same time, the extensive availability and advertising of such products, and especially those with unbalanced nutrient content, challenge efforts to eat healthily^(8–10)^.

Within the food consumer environment, food availability refers to whether a product is present or not within a food store, and usually, food availability is associated with food advertising, i.e. only food items that are present within a food store are advertised^(11)^. Food advertising within food outlets involves messages on packaging or signage, shelf labelling, and product samples and is a dimension of the consumer food environment that is little investigated^(12,13)^. A systematic review of fifty-six primary, quantitative and observational studies, published from 2000 to 2011, concluded that none of the studies mentioned advertising within food retail outlets and the influence of marketing strategies on an individual's food choices^(14)^.

In Brazil, food advertising is recognised as an obstacle to healthy eating by the Dietary Guidelines for the Brazilian Population since it promotes unhealthy foods more frequently than healthy foods^(15)^. Several studies evaluated food advertising on Brazilian television channels^(16,17)^ and on the Internet^(18)^, but there is little evidence about food advertising within Brazilian food outlets. To our knowledge, only one cross-sectional survey conducted in thirteen districts across the city of Sao Paulo in 2010–2011 examined this aspect of the consumer food environment. The study found that signage or promotion of ultra-processed foods (UPF) was uniformly found across stores, while fruit and vegetable (F&V) promotion was thirteen times less likely to be found at locally owned grocery stores and corner stores^(19)^.

As is prevalent worldwide, Brazil faces alarming obesity and NCDs occurrence^(20)^. Consequently, multisectoral actions that promote healthy eating and physical activity have been proposed in the country including the Health Academy Program (PAS: Programa Academia da Saúde). This initiative was created in 2011 and consists of primary healthcare services allocated in socially vulnerable regions that offer physical activity and healthy eating guidance at no cost to its participants. PAS is mainly utilised by older women and individuals who have low levels of education and are overweight^(21)^. Since the food environment can impact individuals’ food choices^(1–4)^, in addition to describing the programme effectiveness in changing its users’ behaviours, it is also interesting to understand how the PAS food environment is characterised. One previous study already showed that the PAS food environment is recognised as adequate in F&V diversity and variety and excessive in UPF^(22)^. The present study intended to advance research on this topic and aimed to describe the advertising and the availability of food items within food outlets around PAS units.

## Methods

The present study was conducted in the neighbourhoods of eighteen PAS units in Belo Horizonte from April to September 2013. The city is the sixth largest in Brazil and the eighth largest in Latin America, with an estimated population of 2 501 576^(23)^.

In the sampling process, 42 of the 50 PAS units installed in the municipality were considered. Six units located in areas of low health vulnerability were excluded due to their reduced number in the municipality. Two additional units were excluded because they have participated in a previous intervention study. Thus, eighteen (42⋅8 %) units distributed through the nine regions of the municipality were randomly assigned, representing the total with a confidence level of 95 and 1⋅4 % error. More information about methods and sampling can be seen in a previous publication^(24)^.

To define the food environment, establishments that traded F&V within a buffer with a 1600-m radius around each PAS unit sampled were selected. Open-air food markets were not included. The food outlets were included in the study if their owners allowed data collection. The ArcView buffer tool (Esri, Redlands, CA, USA) was chosen to create these buffers.

**Fig. 1.**
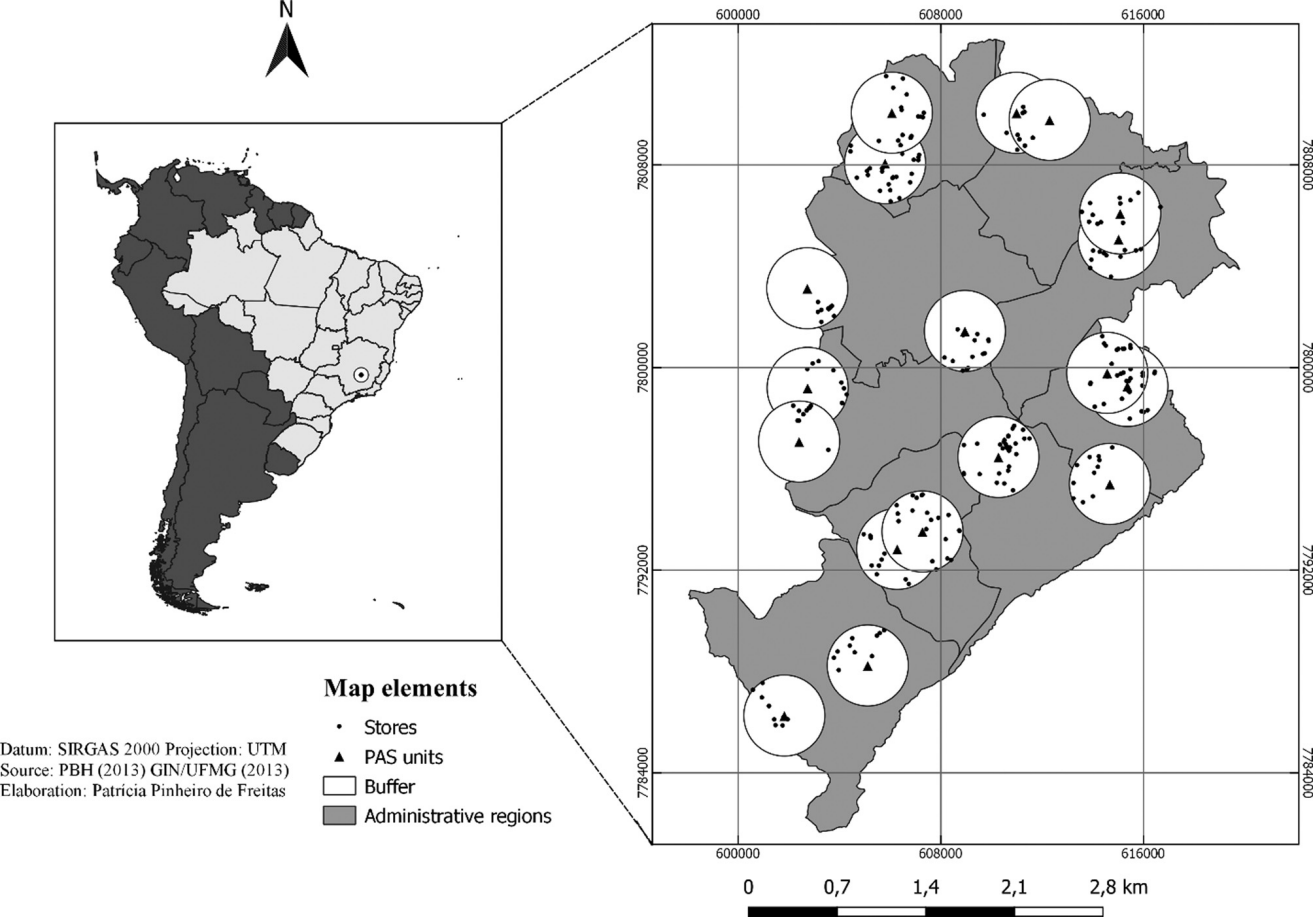
PAS (*Programa Academia da Saúde*) units and food stores selected.

In total, our sample comprised 281 establishments (Fig. 1) which were classified into the following categories: large-chain supermarkets; specialised F&V markets and local grocery stores, convenience stores or bakeries. These food stores were classified in accordance with the following:

- Availability of F&V: among the types most consumed in the municipality^(25)^ – fruits: banana, orange, papaya, watermelon, apple, mango, pineapple, tangerine, grape, melon, pumpkin; vegetables: chayote, tomato, carrot, lettuce, zucchini, cabbage, beetroot, kale, okra.
- Availability of UPF: among the types most consumed by Brazilians^(25)^ – regular soda, fruit-flavoured drinks and juice/nectars with added sugar, cream-filled chocolate cookies and corn chips snacks.
- Food advertising: by checking the presence of signs with nutrition information, signs or other displays that encourage the purchase or the eating of food products, and discounts^(25)^. Food advertisement was analysed separated for only F&V advertising (any type, except tubers, roots and frozen food), only UPF advertising (regular soda, fruit-flavoured drinks and juice/nectars with added sugar, cream-filled chocolate cookies and corn chips snacks), and both F&V and UPF advertising^(25)^.

UPF is defined as industrial formulations that result from a series of industrial processes and their ingredients often include sugar, oils, fats and salt, generally in combination, in addition to substances such as flavours, colours, emulsifiers and sweeteners^(26,27)^. We chose F&V as an indicator of healthy eating because of their high nutrient density, while UPF represented an unhealthy eating indicator by the unbalanced nutrient composition of these products^(26)^.

Food store types were compared in accordance with the availability of F&V, UPF and food advertising by applying the *χ*^2^ test at 5 % significance level (*P*-value < 0⋅05). All analyses were performed with the statistical software package Stata/se version 13⋅0.

The present study was conducted according to the guidelines in the Declaration of Helsinki and all procedures involving human subjects/patients were approved by the Universidade Federal de Minas Gerais Ethics Research Committee (0537.0.0203.000-11) and the Prefeitura Municipal de Belo Horizonte Ethics Research Committee (0537.0.0203.410-11A). Written informed consent was obtained from all subjects/patients.

## Results

The food outlets comprised mainly specialised F&V markets (59⋅4), 79⋅4 and 83⋅3 % of them contained at least eight types of F&V, respectively. UPF availability was seen in 60⋅9 % of establishments (Table 1).

**Table 1.** Characterisation of the food outlets around the PAS (*Programa Academia da Saúde*) units. Belo Horizonte, MG, Brazil, 2013

Food consumer environment	Total sample		Food outlets type						
			Specialised F&V markets (*N* 167)		Large-chain supermarkets (*N* 60)		Local grocery stores, convenience stores or bakeries (*N* 54)		*P*-value
	*N*	%	*N*	%	*N*	%	*N*	%	
Fruit availability									
0–7 types	58	20⋅6	23	13⋅8	11	18⋅3	24	44⋅4	<0⋅001
≥8 types	223	79⋅4	144	86⋅2	49	81⋅7	30	55⋅6	
Vegetables availability									
0–7 types	47	16⋅7	14	8⋅4	9	15⋅0	24	44⋅4	<0⋅001
≥8 types	234	83⋅3	153	91⋅6	51	85⋅0	30	55⋅6	
UPF availability									
No	110	39⋅1	103	61⋅7	1	1⋅7	6	11⋅1	<0⋅001
Yes	171	60⋅9	64	38⋅3	59	98⋅3	48	88⋅9	
Food advertisement									
Absent	168	59⋅8	117	70⋅1	20	33⋅3	31	57⋅4	<0⋅001
Only F&V advertisement	55	19⋅6	43	25⋅8	9	15⋅0	3	5⋅6	
Only UPF food advertisement	49	17⋅4	7	4⋅2	23	38⋅3	19	35⋅2	
F&V and UPF advertisement	9	3⋅2	0	0⋅0	8	13⋅3	1	1⋅9	

F&V: fruit and vegetables; UPF: ultra-processed food.

Food advertisement was absent in 59⋅8 % of food outlets, 19⋅6 % were advertising only F&V and 17⋅4 % were advertising only UPF (Table 1).

Higher F&V availability was noted inside specialised F&V markets and large-chain supermarkets than local grocery stores, convenience stores or bakeries. UPF availability, on the other hand, was less common inside specialised F&V markets (38⋅3 %) (Table 1).

Food advertising was mostly absent inside specialised F&V markets (70⋅1 %). In the presence of food advertising, the promotion of only F&V was more common within specialised F&V markets. However, large-chain supermarkets and local grocery stores, convenience stores or bakeries contained more frequently UPF food advertising isolated: 38⋅3 and 35⋅2 %, respectively (Table 1).

## Discussion

The present study described the availability and advertising of food within food outlets in the neighbourhoods of PAS in Brazil. Specialised F&V markets had a higher availability of F&V and the highest presence of only F&V advertising. Large-chain supermarkets also had high availability of F&V, although predominantly only UPF advertising. Local grocery stores, convenience stores or bakeries had less availability of F&V and a higher presence of UPF advertising.

Specialised F&V markets were the most common food outlets around PAS units and had higher F&V availability and advertising. Belo Horizonte, the metropolis examined in the present study, is an international reference for public policies on food and nutritional security focusing on the implementation of public establishments in deprived and low-income areas to ensure access to healthy eating, such as open-air markets and municipal markets, in addition to the promotion of population health through proper and healthy nutrition^(28)^.

Supermarkets are also singled out as inductors for the consumption of F&V, for presenting a greater variety, better quality and lower cost of these foods^(29)^. However, they are also known for containing high UPF variety and diversity^(29)^. In Brazil, near 60 % of the UPF acquisition is realised in supermarkets^(30)^. These facts can explain the simultaneous occurrence of high UPF availability and advertising and high F&V availability within these food outlets.

In contrast, local grocery stores, convenience stores or bakeries, contained an unhealthy status of food availability and advertising around the PAS units. In line with our findings, these food outlets are known as small food stores that offer an abundance of calorically high, nutrient-low foods. Although they have more autonomy to define the type of food and advertising inside these types of food outlets, in comparison to corporate- and franchise-owned stores, independent stores retailers’ face a more limited infrastructure for offering healthful food and beverages^(29)^.

The novelty of the present study lies in the description of food advertising inside food stores, evidence that is largely scarce in the literature^(12,14)^ and especially in Latin America, where studies of the consumer food environment focus on evaluating and classifying food stores as healthy or unhealthy based on types of foods found in the stores^(31)^.

This knowledge has some significant public health implications since it identifies several public health actions. First, retailers can encourage consumers to choose products by pursuing a range of marketing and merchandising activities and, overall, they demonstrate a willingness to engage in healthy food retail and a desire for greater support from healthy food retail initiatives^(32,33)^. Characterising food outlets based on the availability and advertising of food items can generate actions focusing on stimulating the retailers to improve the healthiness of their establishments. Second, the present study highlights the importance of counselling consumers about the best places to buy foods (i.e. those with higher availability of F&V). Third, to improve PAS effectiveness, actions aiming to improve the retail food environment around these services are also needed. Reducing UPF food advertising inside large-chain supermarkets and local grocery stores, convenience stores or bakeries and substituting it for F&V advertising such as in-store coupons or specials, in-store tastings/recipe demonstrations, and displaying labels or signs on shelves that highlight healthier options are some examples of marketing strategies that attract consumers^(34,35)^.

However, the present study also has several limitations. The audited food stores were located within a defined radius around PAS units; other food environments may influence the participants, although at least three times a week the participants routinely attend these services. In addition, our data are from 2013, and food environments change over time: some stores open and others close, especially in a financial crisis. In the city where the study took place, no dramatic social intervention occurred since the study was conducted and before the recent COVID-19 pandemic. Nevertheless, the present study addresses a major aspect of the consumer food environment that is understudied and practically unknown in Brazil.

## Conclusions

The availability and advertising of food items within food outlets around PAS units are different according to the type of food outlet. In general, specialised F&V markets showed higher F&V advertising. Supermarkets, despite containing high availability of F&V, presented a high occurrence of only UPF advertising. Local grocery stores, convenience stores or bakeries were the food outlets with higher UPF availability and only UPF advertising.
